# Effect of dithiocyano-methane on hexose monophosphate pathway in the respiratory metabolism of *Escherichia coli*

**DOI:** 10.1186/s13568-020-01142-z

**Published:** 2020-11-11

**Authors:** Yanfeng Chen, Wenjie Ke, Huabin Qin, Siwei Chen, Limei Qin, Ying Yang, Hui Yu, Yuansheng Tan

**Affiliations:** Guangdong Provincial Key Laboratory of Animal Molecular Design and Precise Breeding, Key Laboratory of Animal Molecular Design and Precise Breeding of Guangdong Higher Education Institutes, School of Life Science and Engineering, Foshan University, Foshan, 528231 China

**Keywords:** Dithiocyano-methane, Hexose monophosphate pathway, Respiratory metabolism, *Escherichia coli*

## Abstract

This paper studied the inhibitory effects of dithiocyano-methane (DM) on the glucose decomposition pathway in the respiratory metabolism of *Escherichia coli*. We investigated the effects of DM on the activities of key enzymes (ATPase and glucose-6-phosphate dehydrogenase, G6PDH), the levels of key product (nicotinamide adenosine denucleotide hydro-phosphoric acid, NADPH), and gene expression in the hexose monophosphate pathway (HMP). The results showed that the minimum inhibitory concentration (MIC) and the minimum bactericide concentration (MBC) of DM against the tested strains were 5.86 mg/L and 11.72 mg/L, respectively. Bacteria exposed to DM at MIC demonstrated an increase in bacterial ATPase and G6PDH activities, NADPH levels, and gene expression in the HMP pathway compared to bacteria in the control group, which could be interpreted as a behavioral response to stress introduced by DM. However, DM at a lethal concentration of 10 × MIC affected glucose decomposition by inhibiting mainly the HMP pathway in *E. coli*.

## Introduction

Respiratory metabolism of bacteria is an important manifestation of life activities, and its essence is the oxidation and degradation process of intracellular saccharides. The oxidative metabolism of saccharides is vital to provide the energy needed for cellular activities, the carbon scaffolds for anabolism and the metabolism of bacteria. There are three main pathways of glucose decomposition in bacteria: (1) the glycolysis pathway (EMP) under anaerobic conditions in which lactic acid or ethanol are generated from glucose; (2) the tricarboxylic acid cycle pathway (TCA) under aerobic conditions in which the glucose is completely oxidized to water and carbon dioxide; and (3) the hexose monophosphate pathway (HMP) in which glucose is oxidized to supply cell body with NADPH, ribose and its derivatives (e.g. coenzyme).

Pathogenic *Escherichia coli*, a zoonotic pathogen of humans and animals, leads to many diseases in humans, poultry and livestock (Woodward et al. 1990; Boudeau et al. 1999; Frydendahl 2002; Mellata et al. 2003; Kwak et al. 2016), posing a great threat to human health and the development of animal husbandry. Disinfectant is currently the primary method to kill pathogenic *E. coli* in the living environment of humans, poultry and livestock. However, the application of disinfectant to eliminate pathogenic *E. coli* strains isolated from hospitals, communities, livestock and poultry farms has been limited by the growing problem of drug resistance (Li et al. 2012; Chen and Xia 2013; Yang et al. 2015; Ibrahim et al. 2019). Therefore, it is necessary to search for new solutions to prevent *E. coli* infections.

Dithiocyano-methane (DM, Structural formula: SCN-CH_2_-SCN), also known as methylene bisthiocyanate (Singh et al. 2006; Qi et al. 2008) or methylene dithiocyanate (Chen et al. 2003, 2004), has been used as an antifungal agent in plant protection and the aquaculture industry (Chen et al. 2003; Qi et al. 2008; Cao et al. 2012). However, the antibacterial properties of DM against bacteria have been neglected and therefore it has not been widely used in many industries.

The antimicrobial action mechanism of DM has not been fully elucidated. Some studies indicated that DM inhibited the respiration of *Fusarium moniliforme* and *Saccharomyces cerevisiae* by affecting the mitochondrial respiration rate and cytochrome oxidase in the electron transport chain (Chen et al. 2003, 2004). Similar results also suggested the effect of DM on *Ophiostoma floccosum*, which indicated this agent not only inhibited fungal respiratory activity but also reduced ATP level and glucose consumption (Singh et al. 2006). However, no further studies have been carried out on the mechanism of the decrease in microbial glucose consumption in response to DM. This study investigated which glucose decomposition pathway is inhibited and how this pathway is affected in *E. coli* when exposed to DM. The goal of current study is to elucidate the antimicrobial mechanism of DM, investigate the antibacterial effectiveness of DM against *E. coli*, and provide a reference for its application as an antibacterial agent.

## Materials and methods

### Materials

Dithiocyano-methane (DM) was purchased from Guangzhou Kafen Biotechnology Co., Ltd. *Escherichia coli* standard strain CFT073, deposited in the American Type Culture Collection (ATCC 700928), and *E. coli* strain O157, isolated from diseased pigs, were kindly donated by Dr. Liu Canying from Foshan University. Two different strains were used to determine the minimum inhibitory concentration (MIC) and minimum bactericide concentration (MBC), while only strain CFT073 was used to carry out the rest experiments.

The purified strain was maintained at −80 °C in sterile nutrient broth containing 30% glycerol. The bacteria were streaked on nutrient agar plates and incubated at 37 °C for 24 h. A sterilized loop was used to transfer the colonies to sterile nutrient broth in a test tube one day before the experiment. The culture was incubated on a shaker (100 rpm) at 37 °C overnight. Bacteria in the logarithmic growth phase were used in the experiments.

DM of 0.3 g was dissolved in sterile distilled water (100 mL) by slow heating below 70 °C with stirring. After cooling to room temperature, the solution was sterilized by filtration through 0.22 μm filter membranes. The stock solution was stored at 4 °C.

### Determinations of MIC and MBC

The MIC was determined using a two-fold-broth-dilution method by following the National Committee for Clinical Laboratory Standards reference method (NCCLS 2000). Stock solution of DM was added to nutrient broth to obtain final concentrations of 187.5, 93.75, 46.88, 23.44, 11.72, 5.86, 2.93, 1.46, 0.73 and 0.37 mg/L, respectively. *E. coli* CFT073 was incubated and adjusted to 2.95 × 10^8^ CFU/mL. Then 100 μL of bacterial suspension was added to the above-mentioned mixtures with different concentrations in proper order followed by incubation at 37 °C for 24 h. The mixture containing nutrient broth and bacteria without DM was used as the control group. Aliquots of 100 μL of cultures were removed from the nonvisible bacterial growth tubes and then spread on the surface of nutrient agar. The MBC was defined as the lowest concentration of DM, where no growth was observed after the cells were incubated on nutrient agar plates at 37 °C for 48 h (Ma et al. 2010). The same method was used to determine the MIC and MBC of DM against *E. coli* strain O157.

### Respiration inhibition of DM at a lethal concentration of 10 × MIC against *E. coli* CFT073

The respiration inhibition test was carried out according to previous studies with modifications (Guo et al. 2005; Ma et al. 2010; Qu et al. 2015). A mixture containing 1.6 mL of 1% glucose, 14.4 mL of PBS and 4 mL of *E. coli* CFT073 bacterial suspension in the logarithmic growth phase was stirred lightly for 5 min in a reagent bottle sealed with a preservative film. During the experiment, each reagent bottle was continuously stirred. Oxygen consumption was gauged by a JPB-607A dissolved oxygen analyzer (Leici, INESA Scientific Instrument Co., Ltd, Shanghai, China) at 2.5 min intervals for 5 min, obtaining the initial respiratory rate (μmol O_2_ g^−1^ min^−1^). Then, the stock solution of DM was added to the above-mentioned mixture, in which the final concentration of DM was 58.6 mg/L (10 × MIC). According to the respiratory rate of *E. coli* CFT073 before and after DM addition, the respiratory inhibition rate of *E. coli* CFT073 was calculated using the following equation:

$$I_R = \frac{R_0 - R_1}{{R_0}}{ \times }100\%$$

where, *I*_*R*_ is the respiratory inhibition rate of *E. coli* CFT073 after DM addition, and *R*_*0*_ and *R*_*1*_ is the respiratory rate of *E. coli* CFT073 before and after DM addition, respectively.

Three typical inhibitors, iodine acetic acid, malonic acid, and sodium phosphate, were used in the experiment. The inhibitory superposition rate of *E. coli* CFT073 was calculated using the following equation:

$$D_R = \frac{R_1 - R_2}{{R_1}} { \times }100\%$$

where, *D*_*R*_ is the inhibitory superposition rate of *E. coli* CFT073; *R*_*1*_ is the respiratory rate of *E. coli* CFT073 after DM addition; *R*_*2*_ is the respiratory rate of *E. coli* CFT073 after the addition of inhibitor and DM.

### Activities of ATPase and glucose-6-phosphate dehydrogenase (G6PDH) in *E. coli* CFT073 exposed to DM at a concentration of MIC

DM was added to the bacterial suspension of *E. coli* CFT073 in the logarithmic growth phase with the final concentration of MIC. After exposure for 0, 1, 4, 24 h, the activities of ATPase and G6PDH were determined according to the instruction of the test kits (Jiancheng, Nanjing Jiancheng Bioengineering Institute, Nanjing, China & Keming, Suzhou Comin Biotechnology Co., Ltd, Suzhou, China) by ultraviolet spectrophotometer.

### Levels of nicotinamide adenosine denucleotide hydro-phosphoric acid (NADPH) in *E. coli* CFT073 exposed to DM at a concentration of MIC

DM was added to the bacterial suspension of *E. coli* CFT073 in the logarithmic growth phase with the final concentration of MIC. After exposure for 0, 1, 4, 24 h, the levels of NADPH were determined according to the instruction of the test kit (Keming, Suzhou Comin Biotechnology Co., Ltd, Suzhou, China) by ultraviolet spectrophotometer.

### Real-time quantitative PCR (RT-qPCR) analysis of the expression of genes in the HMP pathway and genes related to the stress response

DM was added to the bacterial suspension of *E. coli* CFT073 in the logarithmic growth phase with the final concentration of MIC. After exposure for 0, 1, 4, 24 h, bacterial suspensions were sampled. Total RNA was extracted from the bacterial cells using the EasyPure RNA Kit (TransGen Biotech, Transgen Biotechnology Co., LTD, Beijing, China). Total RNA of 1 µg was used for cDNA synthesis with the PrimeScript™ II 1st Strand cDNA Synthesis Kit (Takara, Takara Biotechnology (Dalian) Co., Ltd, Dalian, China). RT-qPCR was carried out in a 96-well plate using the SYBR ExScript RT-qPCR Kit (Takara, Takara Biotechnology (Dalian) Co., Ltd, Dalian, China) as described previously (Hu et al. 2010). The expression level of genes in Table 1 was analyzed using the comparative threshold cycle method (2^−△△CT^) with 16S rDNA as control. All data are relative mRNA expressed as means ± standard deviations.

**Table 1:** Primers used in this study

Gene	Primer sequences (5′ → 3′)	Accession number in NCBI
Fructose-bisphosphate aldolase (*fbaA*)	(F) AACGTGGTTCTGACTCCGAC (R) GAAGTTCAGGCTGTTGTGCG	NC_000913
Fructose-1,6-bisphosphatase (*fbp*)	(F) TGGATGGCTCGTCCAACATC (R) TATACCACGTAACCTGCCGC	EU890589
Ribose-5-phosphate isomerase (*rpiA*)	(F) GCACACTTTATTGACGCGCT (R) GGCTGTCGACTTCGTTGAGA	NC_011751
6-phosphogluconolactonase (*pgl*)	(F) GATGGTCATCTGGTGGCACA (R) CCCAGACATCCACTGAGCTG	NC_011751
Transketolase A (*tktA*)	(F) TCGACTGAACATAGCGGTCG (R) GACAGTGGCCTGTGCTAACT	JQ582675
Transaldolase A (*talA*)	(F) ACAGCGGCGATATTGAGTCCATTC (R) CTGCGACCACCTGTTGTTCCTG	NC_011750
Triosephosphate isomerase (*tpiA*)	(F) TGGCTGCCGGTATTGGTTAC (R) ATCCGGCATTGGGTTTGACT	EU891919
6-phosphogluconate dehydrogenase (*gnd*)	(F) AGCAACAGATCGGCGTAGTC (R) TCTTCTCACGGGAACGGTTG	M23181
Glucose-6-phosphate dehydrogenase (*zwf*)	(F) TACTTCGAGGAGTGCCAGGT (R) CAGCAGGTGGTTCTGGATCA	EU899540
16S rDNA	(F) CGTCGTAGTCCGGATTGGAG (R) TCACCGTGGCATTCTGATCC	
*rpoS*	(F) GGTCTGGCGTTGCTGGATCTTATC (R) ACCAGGTTGCGTATGTTGAGAAGC	NC_011750
*marA*	(F) CCTGGAATCGCCACTGTCACTG (R) ATCTTACGGCTGCGGATGTATTGG	NC_011750
*soxS*	(F) GCCAACGCCGCCTGTTACTG (R)GAAGGTCTGCTGCGAGACATAACC	NC_011750

### Statistical analysis

All experiments were performed in triplicate or more. Values were expressed as means and standard deviations. Statistical analysis was performed on SPSS 19.0 software, and probability levels of less than 0.05 were considered statistically significant (*P *< 0.05).

## Results

### MIC and MBC of DM against the tested *E. coli* strains

In the present study, the MIC and MBC value of DM against two *E. coli* strains were 5.86 mg/L and 11.72 mg/L, respectively.

### Respiration inhibition of DM at a lethal concentration of 10 × MIC against *E. coli* CFT073

As shown in Table 2, the inhibitory superposition rate of malonic acid, sodium phosphate, iodine acetic acid and DM was (74.82 ± 7.14)%, (30 ± 12.02)% and (60.74 ± 5.59)%, respectively.

**Table 2:** Effect of dithiocyano-methane on the respiration inhibition of *E. coli* CFT073

Inhibitor	*E. coli* CFT073	
	Respiratory inhibition rate (I_R_)	Inhibitory superposition rate (D_R_)
Dithiocyano-methane	42.73 ± 2.39	–
Sodium phosphate	26.11 ± 6.73	30 ± 12.02^a^
Iodine acetic acid	44.81 ± 5.01	60.74 ± 5.59^b^
Malonic acid	55.19 ± 5.01	74.82 ± 7.14^b^

Different letters indicate significant differences among different superpositions (*P *< 0.05)

### Activities of ATPase and G6PDH in *E. coli* CFT073 exposed to DM at a concentration of MIC

**Fig. 1:**
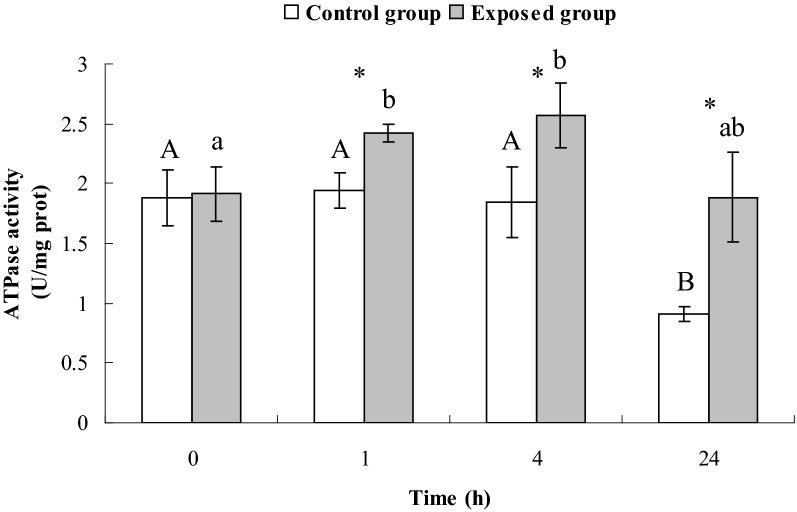
ATPase activities of E. coli CFT073 in control group and exposed group. Different small letters indicate significant differences in the exposed group among different times (P < 0.05); different capital letters indicate significant differences in control group among different times (P < 0.05); the asterisk indicates significant differences between the control group and exposed group at the same moment (P < 0.05), and bars represent standard deviations of the means, the same below

The effects of DM on the ATPase activities of *E. coli* CFT073 are presented in Fig. 1. The ATPase activities in exposed group increased significantly at 1 h and 4 h compared to 0 h (*P* < 0.05). The ATPase activities of *E. coli* CFT073 in exposed group were significantly higher than those in control group at 1 h, 4 h and 24 h (*P* < 0.05).

**Fig. 2:**
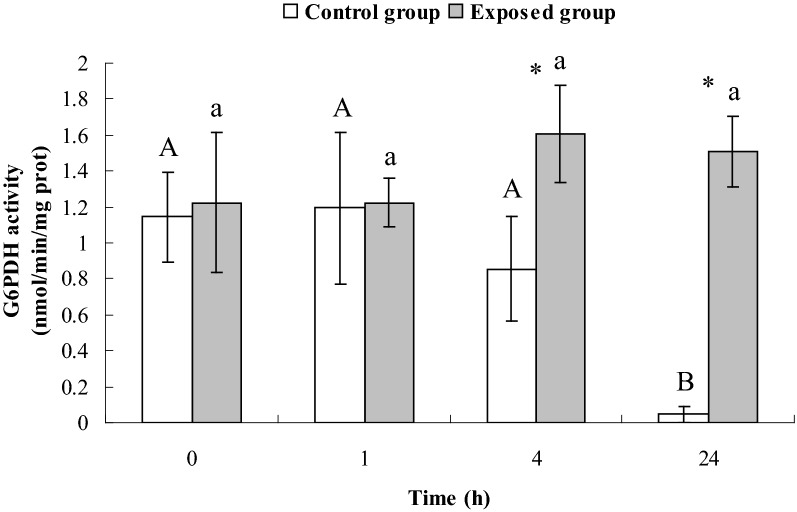
G6PDH activities of E. coli CFT073 in control group and exposed group

As shown in Fig. 2, there were no significant differences in G6PDH activities of *E. coli* CFT073 in exposed group at different times (*P* > 0.05). However, the G6PDH activities in exposed group were significantly higher than those in control group at 4 h and 24 h (*P* < 0.05).

### NADPH levels in *E. coli* CFT073 exposed to DM at a concentration of MIC

**Fig. 3:**
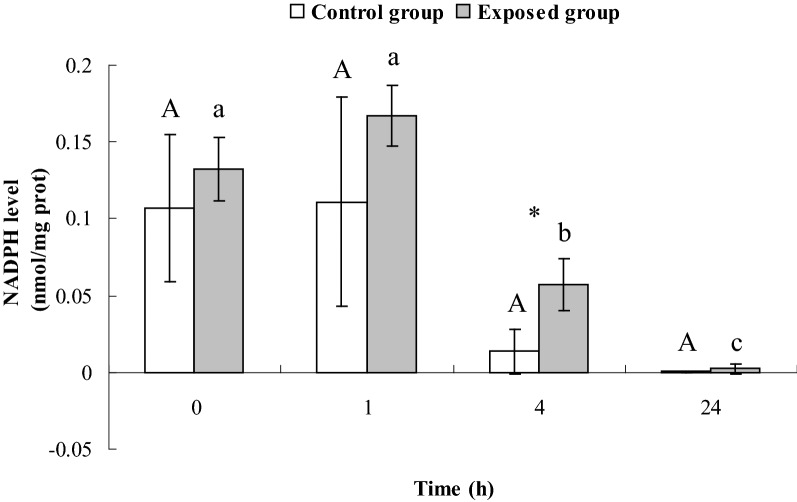
NADPH levels of *E. coli* CFT073 in control group and exposed group

As shown in Fig. 3, the NADPH levels of *E. coli* CFT073 in exposed group at 4 h and 24 h were significantly lower than those at 0 h and 1 h (*P* < 0.05). The NADPH levels in exposed group were significantly higher than those in control group at 4 h (*P* < 0.05). The NADPH levels were very low in both control and exposed groups at 24 h.

### RT-qPCR analysis of the expression of genes in the HMP pathway

**Fig. 4:**
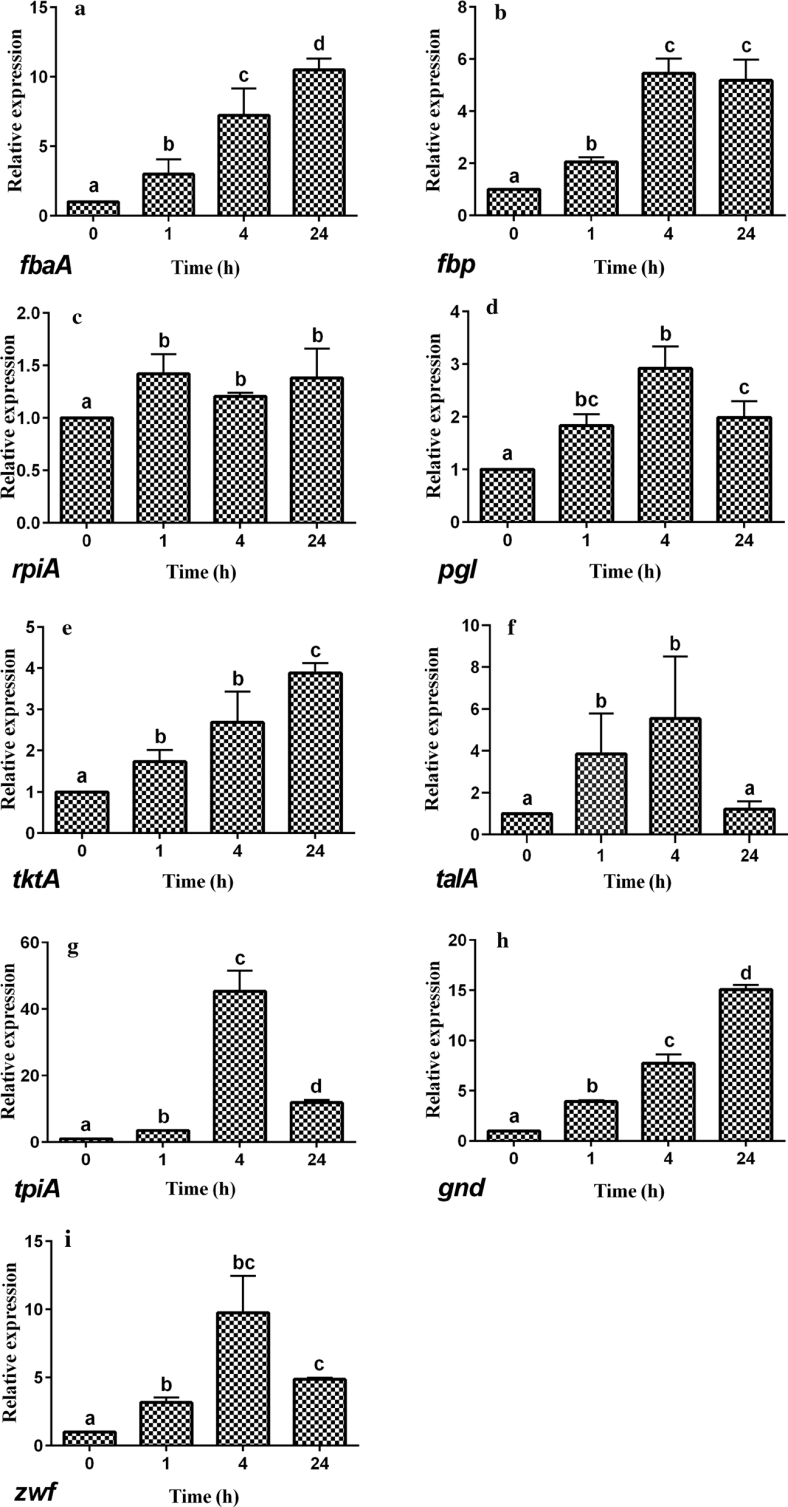
Relative expression of genes in hexose monophosphate pathway of E. coli CFT073 exposed to dithiocyano-methane. Different small letters indicate significant differences in exposed group among different times (P < 0.05), and bars represent standard deviations of the means

The effects of DM on the expression of genes in the HMP pathway of *E. coli* CFT073 are presented in Fig. 4. It showed that DM led to a significant up-regulation of the expression of all genes for 1 h, 4 h and 24 h compared to 0 h. The expression levels of *fbaA*, *tktA* and *gnd* gene were up-regulated gradually after exposure to DM (Fig. 4a, e, h). The expression levels of *pgl*, *talA* and *tpiA* gene were first up-regulated and then down-regulated (Fig. 4d, f, g). In addition, the expression levels of *fbp* and *zwf* gene were up-regulated significantly at 1 h (*P* < 0.05), whereas there were no significant changes in their expression levels after 4 h (*P* > 0.05) (Fig. 4b, i). There was no significant difference in the expression levels of *rpiA* among 1 h, 4 h and 24 h (*P* > 0.05) (Fig. 4c).

### RT-qPCR analysis of the expression of genes related to the stress response

**Fig. 5:**
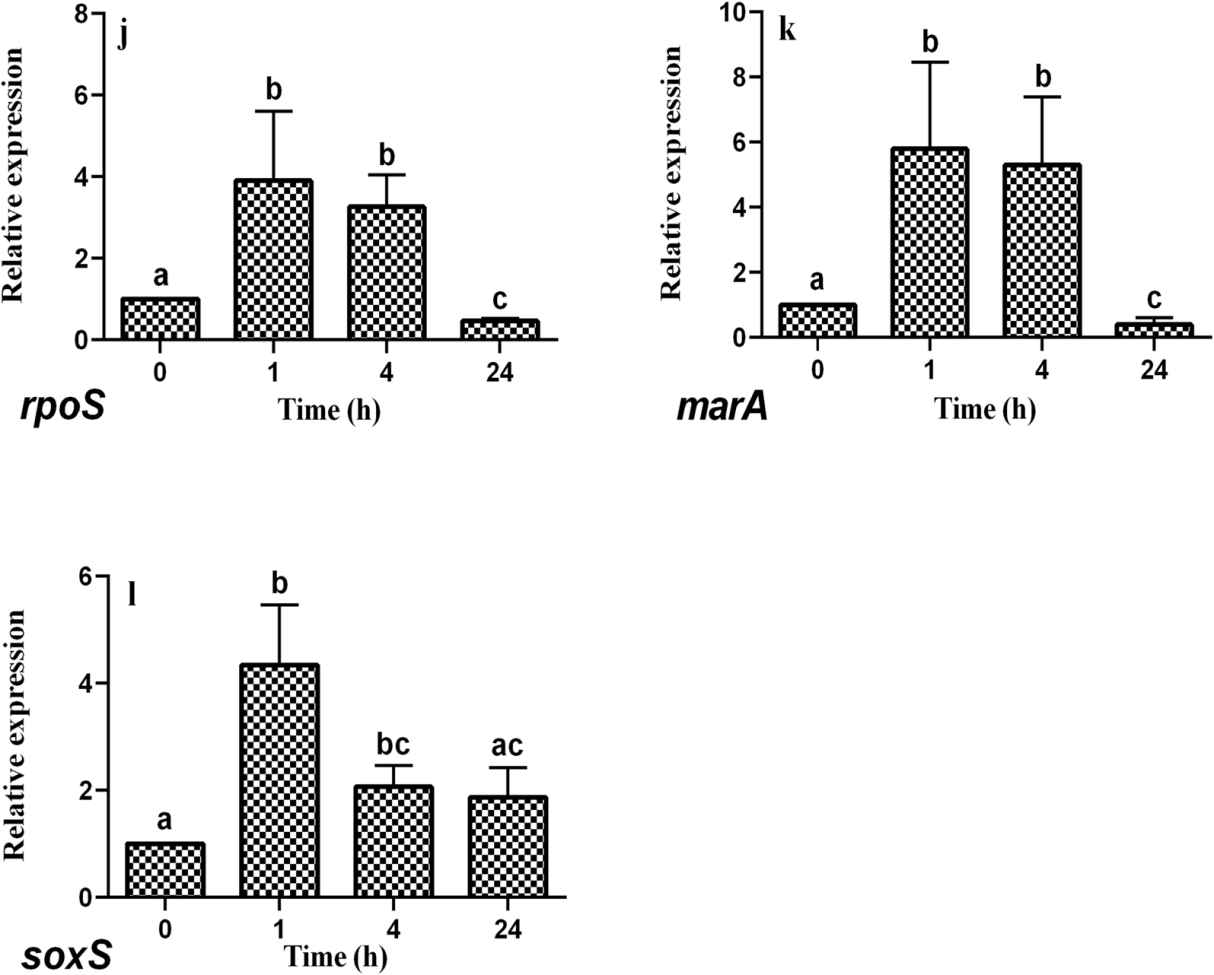
Relative expression of genes related to stress response of E. coli CFT073 exposed to dithiocyano-methane. Different small letters indicate significant differences in exposed group among different times (P < 0.05), and bars represent standard deviations of the means

The results in Fig. 5 show the effects of DM on the expression of genes related to the stress response of *E. coli* CFT073. The expression levels of *marA* and *rpoS* genes were up-regulated significantly at 1 h and 4 h while down-regulated significantly at 24 h compared to 0 h (*P* < 0.05) (Fig. 5 k, j). Furthermore, the expression levels of the *soxS* gene were up-regulated significantly at the 1 h and 4 h (*P* < 0.05), whereas there was no significant difference in the expression levels between 0 and 24 h (*P* > 0.05) (Fig. 5 l).

## Discussion

### Toxicity and stability of DM

Although DM in powder form is harmful to the human body (Braun et al. 2006), the products formed after degrading DM in aqueous solution are non-toxic thiocyanate ions and formaldehyde, and the latter can be further oxidized to carbon dioxide under aerobic conditions (Qi et al. 2006), resulting in low environmental residue (Zhu et al. 1994). There have been some successful cases of DM used in the aquaculture industry in China. DM was used to treat eel (*Anguilla japonica*) skin ulceration disease caused by a pathogenic fungus, *Protoachlya paradoxa*, with a cure rate of 100%. Additionally, no fish fatalities were resulting from DM toxicity, suggesting that DM is harmless to fish at the effective bactericidal concentration (Chen 2010). Another study confirmed that the safe dose of DM for juvenile *Odontobutis potamophila* was higher than the conventional dose, indicating that it could be used in its seed-rearing (Liu et al. 2019).

Zhu et al. (1994) showed that DM was not subject to decomposition below 70 °C, but could be decomposed rapidly above 70 °C. The temperature during the preparation of DM stock solution in this study was below 70 °C.

The spontaneous elimination effect of DM at different pH and temperatures in water has also been investigated (Wu et al. 2015). The results indicated that it was stable under acidic conditions but was easy to decompose above pH 8.5. The available experimental data showed that DM could not be detected after a day in water at pH 8.5 and it could not also be detected after five days in water at pH 8, measured using Ultra Performance Liquid Chromatography method. Furthermore, with the increase of water temperature, the elimination rate of DM increased, and it could not be detected after twelve days in water at 35 °C. Although DM had its limitations since it was only suitable for environments with pH less than 8.5 and it needed to be stored at low temperature, there was evidence that DM could destroy the cellular structure of microorganisms and disturb physiological functions within a few hours (Singh et al. 2006), meaning that its bactericidal capacity was not affected by spontaneous elimination.

### The inhibitory ability of DM against the tested *E. coli* strains

In recent years, studies on the inhibitory ability of DM against microorganisms have been focused on the inhibitory effect of this agent against pathogenic fungi in vitro. The effective inhibitory concentration of DM on mycelial growth, sporangia formation, zoospores release and germination of pathogenic *Saprolegnia* sp*.*, isolated from the crucian carp eggs, was 1 mg/L, 0.5 mg/L, 1 mg/L and 0.5 mg/L, respectively (Xia 2011). Another study showed that the MIC value of this agent against another strain of *Saprolegnia* sp*.* from diseased sturgeons was 6.25 mg/L (Zhang 2011). DM, at a concentration range of 0.25–1.25 mg/L, significantly inhibited the production of zoospore of *Saprolegnia ferax* (Cao et al. 2012).

However, to our present knowledge, the antibacterial effect of DM on bacteria is unclear. In the present study, DM showed remarkable antibacterial activity against the tested *E. coli* strains, which provided a reference for its application as a new efficient disinfectant to the prevention of *E. coli* infections, especially when faced with increasingly severe bacterial resistance to common disinfectants.

### DM at a lethal concentration of 10 × MIC inhibited mainly the pathway of glucose decomposition of *E. coli* CFT073

The current study demonstrated the respiration inhibition rate of DM to *E. coli* CFT073 was (42.73 ± 2.39)%. The O_2_ consumption of the tested strain decreased after exposed to DM. Singh et al. (2006) also reported that the rate of O_2_ consumption of fungus *O. floccosum* reduced when exposed to DM at concentrations higher than 1 mmol/L. Furthermore, DM inhibited the respiration of some fungus such as *Saccharomyces cerevisiae* by affecting the function of mitochondria and the activity of NADH oxidase (Chen et al. 2004). Fungal respiration depends mainly on mitochondria, but bacteria are lack of such organelle. These results suggest DM inhibits fungal and bacterial respiration via a different mechanism.

Iodine acetic acid, malonic acid and sodium phosphate is the typical respiratory inhibitor of the EMP pathway, TCA pathway and HMP pathway, respectively (Guo et al. 2005; Ma et al. 2010; Qu et al. 2015). If two inhibitors block the same pathway, the superposition of the inhibitory rate is weak. On the other hand, if they block different pathways, the superposition increases significantly. The result of the respiration inhibition test in this study indicated that DM at a lethal concentration of 10 × MIC inhibited mainly HMP pathway. Studies have shown that HMP pathway in *E. coli* not only plays a role in the decomposition of glucose but also provides cells with many intermediates for the anabolism, including amino acids, nucleotides and polysaccharides (Sprenger 1995; Zhao et al. 2004). If this pathway is blocked, the production of essential metabolites for biosynthesis in bacterial cells will be inhibited, resulting in the physiological disorder of bacteria (Additional file 1) (Wang et al. 2002).

### Potential correlation among ATPase, G6PDH, NADPH and the effect of DM at a concentration of MIC on them

It was reported that a transformation relationship between two nicotinamide adenine dinucleotides existed in liver mitochondria of rats (Kaplan et al. 1956), as shown in the following equation:

$$NADPH + NAD^{ + } \rightleftharpoons NADP^{ + } + NADH$$

During ATP hydrolysis by ATPase, the equilibrium in above equation strongly shifted to the left (Drachev et al. 1980), resulting in an increase of NADPH. These studies revealed an indirect relationship between ATPase and NADPH in mammals. In addition, the transformation relationship represented by the above equation was also found in *Rhodospirillum rubrum* (Kondrashin et al. 1980). However, it is unclear whether such a relationship between ATPase and NADPH also exists in *E. coli*.

A previous study demonstrated that ATPase activities in red blood cell membranes of patients with G6PDH deficiency were lower than those of normal people (Jiang et al. 1991), indicating a relationship between ATPase and G6PDH in the human body. In our study, both G6PDH activities and ATPase activities in the exposed group did not change significantly between 1 and 24 h, supporting this proposed relationship between ATPase and G6PDH activity.

Four hours after exposure, both the activity of G6PDH and the levels of NADPH in the exposed group were significantly higher than those in control group (Figs. 2 and 3). This is likely because bacteria were in the logarithmic growth phase with sufficient glucose in the medium, so glucose metabolism was active in the bacteria. After 24 h of exposure, G6PDH activity remained higher in the exposed group than the control group. However, there were lower levels of NADPH (Figs. 2 and 3). At this time, the bacteria were in the decline growth phase with a depleted supply of glucose in the medium and could only produce a limited amount of NADPH. Subsequently, NADPH was consumed to maintain the metabolic activity of bacteria in response to the DM insult, leading to a decrease in NADPH level.

In the HMP pathway, NADPH competes with NAPD^+^ to bind to the active site of G6PDH. Therefore, G6PDH activity is directly affected by the ratio of NAPD^+^/NADPH. In the present study, after exposure to DM at a concentration of MIC, NADPH level in the exposed group at 24 h significantly decreased compared to 1 h, promoting G6PDH activity. However, once NADPH became excessive, it in turn inhibited G6PDH activity. This might explain our result that G6PDH activities in the exposed group did not change a lot between 1 and 24 h.

The primary function of ATPase in living organisms is to catalyze the hydrolysis of ATP to form ADP and phosphate (Pi) and release energy (Bolognani et al. 1993). The increased ATPase activities observed in this study could indicate an increase in ATP hydrolysis. The activation of the HMP pathway by DM at a concentration of MIC would further consume more ATP in order to catalyze glucose to form more glucose-6-phosphate using hexokinase. The combination of the above two reactions resulted in an increase in ATP consumption. This result is in line with the finding by Singh et al. (2006) showing the ATP level in microbial cells reduced after exposure to DM.

### Reasons for the increase of G6PDH activities, NADPH level and gene expression levels in HMP pathway after exposure to DM at a concentration of MIC

In the present study, DM at a lethal concentration of 10 × MIC inhibited the HMP pathway in the respiratory metabolism of *E. coli.* In contrast, DM at a concentration of MIC activated this pathway, increasing bacterial key enzyme activities and gene expression levels. This observation is generally consistent with a previous study showing that DM at low concentrations had little effect on fungal respiration, but inhibited it at high concentrations (Singh et al. 2006).

Iida et al. (1993) showed that there were two transketolases (*tktA* and *tktB*) responsible for erythrose 4-phosphate (E4P) synthesis. Transketolase A encoded by *tktA* plays a significant role in E4P production in the HMP pathway. Overexpression of *tktA* increases the yield of E4P (Tatarko and Romeo 2001), which is essential for the biosynthesis of aromatic amino acids, such as phenylalanine, tyrosine and tryptophan. The main metabolic pathway of phenylalanine is to produce tyrosine via hydroxylation. And tyrosine undergoes a series of catabolism to form fumaric acid to enter the tricarboxylic acid cycle. In addition, acetoacetyl coenzyme A, one of the end products of tryptophan catabolism, is formed by thiolase, which indicates a link between aromatic amino acids and tricarboxylic acid cycle. Moreover, the transketolase in the HMP pathway is also closely related to the production of intermediates such as ribose 5-phosphate and heptanose 7-phosphate. The former is employed for the biosynthesis of purine and pyrimidine nucleotides, and the latter is utilized for the biosynthesis of bacterial cytoderm components (Iida et al. 1993; Maifiah et al. 2017). In summary, the up-regulation of *tktA* gene expression was presumably due to the accelerated biosynthesis of the essential substances to maintain the physiological functions in *E. coli* in response to the stress induced by DM insult. It was reported the *E. coli rpoS* gene encoded a sigma factor, RpoS, which was required for an expression of a large number of genes in response to various stresses (Stoebel et al. 2009). In *E. coli*, *rpoS* positively regulated the expression of *tktA* gene in the exponential growth phase, but negatively in the stationary growth phase (Jung et al. 2005). Our study showed that the stress in response to DM insult led to the up-regulation of *rpoS* expression in *E. coli* in the exponential growth phase (1 h and 4 h), and subsequently, the up-regulation of *tktA* expression. When the expression of *rpoS* in *E. coli* in the stationary growth phase declined at 24 h after exposure, *tktA* expression remained increased.

G6PDH played an essential role for *E. coli* to survive the stress induced by antibacterial agents. It has been demonstrated that the levels of G6PDH increased by the induction of *zwf* gene after cells were exposed to paraquat (Niazi et al. 2007). Tellurite-exposed *E. coli* also exhibited increased *zwf* expression, G6PDH activity and NADPH level (Sandoval et al. 2011, 2015), which was in line with the results in the present study. MarA and SoxS in *E. coli*, members of the AraC transcriptional activator family, also responded to stimuli such as the presence of antibacterial agents (Duval and Lister 2013). In *E. coli*, transcription of *zwf* gene was activated by MarA or SoxS in response to stress conditions (Jair et al. 1995; Fawcett and Wolf 1995). These data validate the up-regulation of *tktA* and *zwf* expression in *E. coli* CFT073 after exposed to DM in our study.

In summary, the low MIC and MBC of DM in this study demonstrates DM as an effective bactericidal agent and its potential in preventing pathogenic *E. coli* infections. Unique properties of DM aqueous solution, including its effectiveness as a bactericide, its degradability and its low residue in the environment, indicate that it can potentially be used as a disinfectant in settings such as hospitals, communities and farms.

## Supplementary information

**Additional file 1.** Schematic diagram of the hexose monophosphate pathway.

## Data Availability

All relevant data are within the manuscript.

## Abbreviations

DM: Dithiocyano-methane
*E. coli*: *Escherichia coli*
G6PDH: Glucose-6-phosphate dehydrogenase
NADPH: Nicotinamide adenosine denucleotide hydro-phosphoric acid
RT-qPCR: Real-time quantitative polymerase chain reaction
MIC: Minimum inhibitory concentration
MBC: Minimum bactericide concentration
HMP: Hexose monophosphate pathway
