# Supplementary material for: Effect of dithiocyano-methane on hexose monophosphate pathway in the respiratory metabolism of Escherichia coli
Source: AMB Express. 2020 Nov 11;10:205. doi: 10.1186/s13568-020-01142-z (PMC7658277; doi:10.1186/s13568-020-01142-z)
Supplement: Supplementary file 1 — Additional file 1. Schematic diagram of the hexose monophosphate pathway. [file 13568_2020_1142_MOESM1_ESM.doc]

Glucose

Hexokinase

Glucose-6-phosphate dehydrogenase (*zwf*)

NADP^+^

ADP

Glucose-6-phosphate

ATP

6-phosphoglucono-δ-lactone

Ribulose 5-phosphate

6-phosphogluconate

6-phosphogluconolactonase (*pgl*)

NADPH+H^+^

H_2_O

H^+^

NADP^+^

CO_2_+NADPH+H^+^

6-phosphogluconate dehydrogenase (*gnd*)

Ribose-5-phosphate isomerase (*rpiA*)

Ribose 5-phosphate

Xylulose 5-phosphate

Xylulose 5-phosphate

Transketolase (*tktA*)

Glyceraldehyde 3-phosphate

Sedoheptulose 7-phosphate

＋

Fructose 6-phosphate

＋

Erythrose 4-phosphate

Transketolase (*tktA*)

Fructose 6-phosphate

Glyceraldehyde 3-phosphate

＋

Dihydroxyacetone phosphate

Fructose-1, 6-diphosphate

Triosephosphate isomerase (*tpiA*)

Fructose-bisphosphate aldolase (*fbaA*)

Fructose-1,6-bisphosphatase (*fbp*)

Fructose 6-phosphate

Glucose-6-phosphate

Schematic diagram of the hexose monophosphate pathway
